# Infliximab Trough Levels and Quality of Life in Patients with Inflammatory Bowel Disease in Maintenance Therapy

**DOI:** 10.1155/2018/1952086

**Published:** 2018-05-08

**Authors:** Rogério S. Parra, Marley R. Feitosa, Letícia C. H. Ribeiro, Lais A. Castro, José J. R. Rocha, Omar Féres

**Affiliations:** Department of Surgery and Anatomy, Ribeirão Preto Medical School, University of São Paulo, Ribeirão Preto, SP, Brazil

## Abstract

**Objective:**

Investigate the association between infliximab trough levels and quality of life in inflammatory bowel disease patients in maintenance therapy.

**Methods:**

We carried out a transversal study with inflammatory bowel disease patients in infliximab maintenance therapy. Infliximab trough levels were determined using a quantitative rapid test. Disease activity indices (partial Mayo Score and Harvey-Bradshaw Index) and endoscopic scores (endoscopic Mayo Score or Simple Endoscopic Score in Crohn's disease) were obtained. Quality of life was assessed using the Inflammatory Bowel Disease Questionnaire (IBDQ).

**Results:**

Seventy-one consecutive subjects were included in the study (55 with Crohn's disease and 16 with ulcerative colitis). Drug levels were considered satisfactory (≥3 *μ*g/mL) in 28 patients (39.4%) and unsatisfactory (<3 *μ*g/mL) in 43 (60.6%). Satisfactory trough levels were associated with higher rates of clinical remission and mucosal healing. Higher trough levels were also associated with improved IBDQ scores, particularly regarding bowel symptoms, systemic function, and social function.

**Conclusion:**

Satisfactory trough levels of infliximab were associated with higher rates of clinical remission, mucosal healing, and improved quality of life in inflammatory bowel disease patients on maintenance therapy.

## 1. Introduction

Inflammatory bowel diseases (IBD), such as Crohn's disease (CD) and ulcerative colitis (UC), constitute a significant burden for the patients and the society [1]. Patients with CD and UC require frequent outpatient care, hospitalizations, and surgeries; some of them need stomas. The described changes cause functional impairment and great deterioration in quality of life. IBD has a chronic character and affects mostly young people. The disease can reduce the ability to work, decrease productivity, cause interruptions in employment, and have negative effects in patients' social and emotional well-being [2].

Infliximab (IFX), an antitumor necrosis factor alpha (TNF-*α*) antibody, is a biologic drug used for the treatment of CD and UC. IFX is effective in inducing and maintaining remission in patients with CD and UC [3]. Despite its effectiveness in both induction and maintenance of remission, a substantial number of patients will eventually lose response. Loss of response (“flares”) is associated with hospitalization and even surgeries, decreasing the quality of life [4, 5].

There is increasing evidence that clinical remission (CR) and mucosal healing (MH) are associated with better response to treatment, less hospitalization, lower surgery rates, and, consequently, improved quality of life in patients with IBD [4–7]. IFX trough levels higher than >2–3 *μ*g/ml are associated with higher rates of sustained clinical and biochemical remission and improved endoscopic outcomes. Patients with relapse often have lower trough serum drug levels [8, 9].

Few studies correlated IFX trough levels with quality of life. The impact of loss of quality of life and, consequently, less social function, emotional functional, and worse symptoms, has not been investigated in detail in our country. The purpose of the present study was to investigate the association between IFX trough levels, response to treatment, and quality of life in a group of Brazilian IBD patients.

## 2. Materials and Methods

### 2.1. Study Design and Participants

A transversal study was performed in 71 consecutive patients with CD and UC on IFX maintenance therapy (5 mg/kg). All of the patients signed an informed consent statement prior to their participation, and the study was approved by the hospital's ethics committee.

### 2.2. Determination of Infliximab Serum Levels

IFX trough levels were obtained from blood samples collected immediately before drug infusion. Quantitative determination in serum was performed with the Quantum Blue® Infliximab assay (BÜHLMANN, Schönenbuch, Switzerland), as described elsewhere [10]. Briefly, serum samples were diluted 1 : 20 and a 70 *μ*L aliquot was loaded into the port of the test cartridge. After a 15 min reaction, the cartridge was read and the results were shown on the point-of-care QB reader display. According to the manufacturer, this kit has the following analytical characteristics: the limit of detection is 0.15 *μ*g/mL, and the lower and upper limits of quantification are 0.4 *μ*g/mL and 20 *μ*g/mL, respectively.

### 2.3. Definition of Clinical Remission and Mucosa Healing

CR was assessed with Harvey Bradshaw Index (HBI) and partial Mayo Score for CD and UC, respectively. In CD, CR was defined as HBI ≤ 4. In UC, CR was defined as partial Mayo Score ≤ 2 (with no individual subscore > 1). Elective ileocolonoscopies/sigmoidoscopies performed at least three months before and three months after blood collection were considered eligible for endoscopic evaluation. MH was considered Simple Endoscopic Score ≤ 2 (in CD) or a Mayo Endoscopic Subscore ≤ 1 (in UC). Levels of infliximab were divided into two categories: satisfactory (≥3 *μ*g/mL) and unsatisfactory (<3 *μ*g/mL) in comparison with MH, CR, and quality of life.

### 2.4. Questionnaires

Quality of life was measured using the Inflammatory Bowel Disease Questionnaire (IBDQ). It consists of a 32-item questionnaire of four dimensions: bowel-related symptoms (e.g., loose stools and abdominal pain), systemic function (e.g., fatigue and sleep pattern), social function (e.g., ability to attend work and social events), and emotional status (e.g., anger, depression, and irritability). The response for each question ranges from 1 (significant impairment) to 7 (no impairment) [11].

The total IBDQ ranges from 32 (very poor quality of life) to 224 (perfect quality of life). Patients in symptomatic remission usually have a score of 170 or more. The validity, reliability, and responsiveness of this questionnaire have been previously established [12].

IBDQ was correlated with CR and MH and with IFX serum levels.

### 2.5. Statistical Analysis

Categorical variables were expressed as frequencies/percentages and continuous variables as means ± standard deviation. The one-sample Kolmogorov-Smirnov test was used to assess the normality of continuous variables. The ANOVA test was used to compare continuous variables. Fisher's exact test or *χ* ^2^ were used to compare categorical variables. The correlation between IFX trough levels, CR, MH, and quality of life was performed through a receiver operating characteristic curve (ROC) analysis. All *p* values were 2-sided, and a significance level of 5% was established. Statistical analysis was performed with IBM® SPSS® Statistics 20 (IBM SPSS, Costa Mesa, CA).

## 3. Results

**Figure 1:**
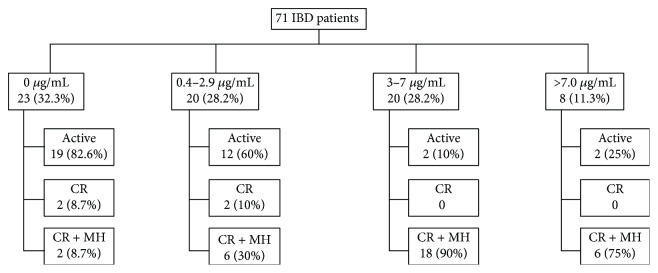
Inflammatory bowel disease (IBD) patients according IFX trough levels and comparison between clinical remission (CR) and mucosal healing.

Seventy-one patients were included (55 with CD and 16 with UC) (Figure 1). Most patients were female (43/60.6%), of Caucasian ethnicity (63/88.7%), economically active (48/67.6), and with higher level of education (52/73.2%).  Table 1 summarizes patients' characteristics according to IFX trough levels. The two groups (satisfactory versus unsatisfactory) were homogeneous. There was no statistical difference between the groups when we compared age, gender, ethnicity, occupation, education, condition (CD versus UC), IFX duration therapy, smoking, and use of azathioprine (mono versus combo therapy).

Of the 71 patients included, 43 (60.5%) have unsatisfactory IFX trough levels, 35 (49.3%) were with active disease, 4 (5.6%) were in CR (but not in MH), and 32 (45%) were in CR and MH (Figure 1).

Satisfactory trough level (≥3 *μ*g/mL) was associated with CR and MH (*p* < 0.001). Similarly, quality of life was better if IFX the trough concentration was higher than ≥3 *μ*g/mL (Table 2).

## 4. Discussion

In this study, we showed that IBD patients with adequate serum IFX levels have higher rates of CR and MH and improved quality of life.

This is the first Brazilian study published to compare quality of life and IFX trough levels. CD and UC have a substantial negative influence on patients' reported quality of life. Patient-reported outcomes are considered an important component of the overall evaluation of treatment effects in patients with IBD [13, 14].

Patients without clinical improvement persistently have impaired quality of life over time. On the other hand, quality of life enhances in patients who regain CR and/or achieve MH. It is known that about half of all IBD patients have relapse in disease activity during IFX maintenance therapy [8, 15]. One of the reasons for loss of response, and consequently decrease in quality of life, is reduction in serum levels of IFX in maintenance therapy. Therapeutic drug monitoring (TDM) could be a useful tool in this cases, allowing physicians to act before loss of response.

TDM is not routinely implemented in our country. However, TDM has shown to improve treatment outcomes and has important economic consequences in patients with IBD [8, 15]. Individualized therapy based on immunopharmacological evidence results in similar CR rates compared to dose intensification strategies. However, the treatment cost is higher when the physician does the “blinded” optimizations, instead of that based on the patient's IFX trough level.

The IBDQ is a validated and reproducible instrument, which can evaluate the quality of life of Brazilian inflammatory bowel disease patients. It includes 32 most frequent and important questions regarding the quality of life in IBD patients and is divided into four areas: intestinal symptoms, systemic symptoms, social aspects, and emotional aspects. This instrument provides additional information that is not evaluated by disease activity indices. Moreover, it is important to see the patient's point regarding his illness, including physical, physiological, and social performance [16–18]. Patient-reported outcomes are considered an important component of the overall evaluation of treatment effects in patients with IBD [19].

Our study showed better quality of life in patients with adequate serum levels of IFX. The patients presented improvement in the intestinal symptoms, besides improvement in the social function and systemic manifestations. Previous studies showed that the activity of CD is an important determinant of better quality of life. To our knowledge, this the first study, in our country, correlating IFX pharmacokinetics and quality of life in patients with IBD. Adequate IFX trough levels were correlated with reduction of signs and symptoms (CR and MH) and consequently better quality of life.

One of the main objectives of disease management is to achieve long-term remission (deeply sustained remission) and prevention of complications, such as fistulas, hospitalizations, surgeries, and stomas, as well as to improve quality of life [2, 19, 20]. It is important to evaluate IBD not only for the symptoms but also for the patients' perspective regarding their disease. Achieving good quality of life in chronic diseases such as CD and UC is a huge challenge for physicians.

Some important study limitations should be noted. First, despite using a validated questionnaire, only one score of quality of life was used. It may be interesting to apply other questionnaires in future studies. Second, this was a transversal study, and the IBDQ was applied in a single opportunity. It would be important to evaluate the patient's quality of life over time. Third, we did not correlate IFX trough levels with proinflammatory biomarkers (such as CRP and faecal calprotectin) as well with the patient's body mass index (BMI). Factors such as BMI and proinflammatory biomarkers may influence IFX trough levels. Our intention is to conduct a prospective study to increase the casuistry and correlate all these parameters. Finally, endoscopic evaluation was not accessed in all patients.

## 5. Conclusions

Adequate IFX trough levels were associated with significantly higher rates of CR, MH, and quality of life in patients with IBD. Achieving quality of life in patients with IBD is a huge challenge for physicians. IBD symptoms have a major impact on patients' lives. TDM can be a useful and important tool in patients losing response. Further prospective studies are needed to confirm our results.

## Figures and Tables

**Table 1:** Patients' main characteristics according to infliximab levels.

Characteristics	TL < 3 *μ*g/mL		TL ≥ 3 *μ*g/mL		*p* value
	*n*	%	*n*	%	
*Age*					
≤40 years	29	61.7	18	38.3	0.803
>40 years	14	58.3	10	41.7	
*Gender*					
Male	23	53.5	20	46.5	0.146
Female	20	71.4	8	28.6	
*Ethnicity*					
White	36	57.1	27	42.9	0.135
Non-white	7	87.5	1	12.5	
*Education*					
Low	11	57.9	8	42.1	0.790
High	32	61.5	20	38.5	
*Occupation*					
Unemployed	17	73.9	6	26.1	0.128
Employed	26	54.2	22	45.8	
*Condition*					
Crohn's disease	35	64.8	19	35.2	0.257
Ulcerative colitis	8	47.1	9	52.9	
*IFX duration*					
≤2 years	20	57.1	15	42.9	0.631
>2 years	23	63.9	13	36.1	
*Azathioprine*					
No	26	57.8	19	42.2	0.618
Yes	17	65.4	9	34.6	
*Smoking*					
No	41	59.4	28	40.6	0.515
Yes	2	100	0	0	

**Table 2:** Clinical remission, mucosal healing, and quality of life scores according to infliximab levels.

Disease control	TL ≥ 3 *μ*g/mL		TL < 3 *μ*g/mL		*p* value
	*n*	%	*n*	%	
Clinical remission	24	85.7	12	27.9	<0.001
Mucosal healing	24	85.7	8	18.6	<0.001

Quality-of-life score	Mean	±SD	Mean	±SD	

Bowel symptoms (maximum = 70)	59.6	9.3	52.3	8.5	0.001
Systemic function (maximum = 35)	27.3	5.6	22.7	5.2	0.001
Social function (maximum = 35)	30.8	5.7	26.7	7.4	0.015
Emotional status (maximum = 84)	65.1	16.5	60.0	12.9	0.148
Global (maximum = 224)	183.0	32.6	161.9	28.9	0.006

## Data Availability

Data were collected by the computerized hospital system and medical records. All data used and analyzed during the current study can be sent, if necessary, as e-mail request (corresponding author). All data analyzed during this study are included in this published article.

## Abbreviations

IFX: Infliximab
IBD: Inflammatory bowel disease
UC: Ulcerative colitis
CD: Crohn's disease
CRP: C-reactive protein
CR: Clinical remission
MH: Mucosal healing
TDM: Therapeutic drug monitoring
IBDQ: Inflammatory bowel disease questionnaire
HBI: Harvey Bradshaw Index
TNF-*α*: Antitumor necrosis factor alpha.

## Supplementary Materials

Supplementary Materials ROC curves for (A) clinical remission and (B) mucosal healing according to infliximab trough levels.
