# Supplementary figures and images for: Infliximab Trough Levels and Quality of Life in Patients with Inflammatory Bowel Disease in Maintenance Therapy
Source: Gastroenterol Res Pract. 2018 May 8;2018:1952086. doi: 10.1155/2018/1952086 (PMC5964568; doi:10.1155/2018/1952086)

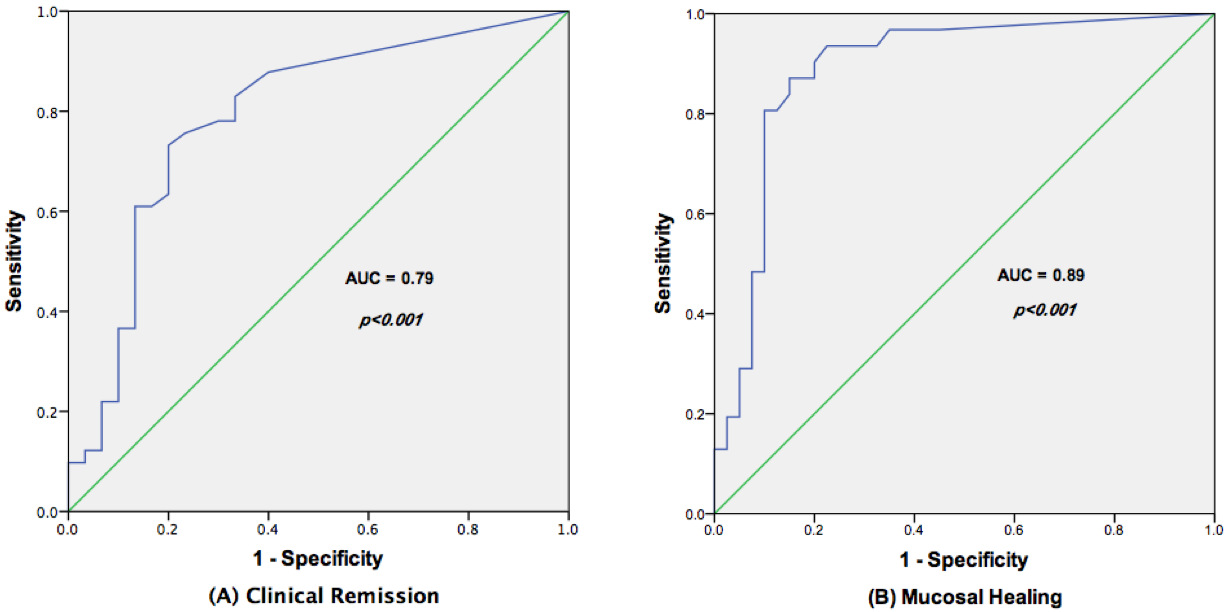

Supplement: Supplementary Materials — ROC curves for (A) clinical remission and (B) mucosal healing according to infliximab trough levels. [file 1952086.f1.png]
